# Prediction of cardiovascular diseases mortality- and disability-adjusted life-years attributed to modifiable dietary risk factors from 1990 to 2030 among East Asian countries and the world

**DOI:** 10.3389/fnut.2022.898978

**Published:** 2022-10-17

**Authors:** Nawsherwan, Wang Bin, Zhang Le, Sumaira Mubarik, Guo Fu, Yan Wang

**Affiliations:** ^1^Xiamen Cardiovascular Hospital of Xiamen University, School of Medicine, Xiamen University, Xiamen, China; ^2^Department of Epidemiology and Biostatistics, School of Public Health, Wuhan University, Wuhan, China; ^3^State Key Laboratory of Cellular Stress Biology, Innovation Center for Cell Signaling Network, School of Medicine, Xiamen University, Xiamen, China

**Keywords:** dietary risk factors, IHD trend, IS trend, prediction, ARIMA model

## Abstract

**Background:**

Unhealthy eating habits are a significant modifiable risk factor for cardiovascular diseases (CVDs); nevertheless, no evidence of their impact on the CVD burden has been reported in East Asian countries. We aimed to determine the trend and predict the future CVDs burden attributed to modifiable dietary risk factors in the East Asian countries (China, Japan, South Korea, and North Korea) and the world.

**Methods:**

The Global Burden of Disease (GBD) 2019 data were used to find the trend of CVDs [ischemic heart disease (IHD) and ischemic stroke (IS)] mortality- and disability-adjusted life-years (DALYs) attributed to dietary risk factors in the East Asian countries and the world (1990–2019) and its prediction from 2020 to 2030. We used the joinpoint regression model and the autoregressive integrated moving average (ARIMA) model for trend and future forecast, respectively.

**Results:**

From 1990 to 2019, regardless of sex, the age-standardized mortality rate (ASMR) and DALYs of IHD attributed to dietary risk factors significantly decreased in Japan, South Korea, and the world. However, the ASMR of IHD significantly increased in Chinese males and for both sexes in North Korea. The ASMR and DALYs of IHD and IS due to dietary risk factors were higher in males than in females in the world. From 2020 to 2030, the ASMR of IHD is predicted to increase in South Korean females and Japanese males. Globally, a diet low in whole grains was the top risk factor for the highest IHD mortality and DALYs in 2019, followed by a diet low in legumes and a diet high in sodium. A diet low in whole grains, a diet high in sodium, and a diet low in legumes were the leading risk factors for high IHD mortality in East Asian countries.

**Conclusion:**

The trend of IHD and IS burden due to dietary risk factors varies substantially across the East Asian countries compared to the trend of CVDs burden in the world. The study findings may help the public health policymakers to design proper strategies for improvement of the quality of life to combat the CVDs burden in the future for the East Asian countries.

## Introduction

Cardiovascular diseases (CVDs) are the leading cause of premature mortality and morbidity among non-communicable diseases (NCDs) worldwide (1). CVDs refer to a class of diseases that involves the heart and blood vessels, including IHD, IS, heart failure, hypertensive heart disease, and several other vascular and cardiac problems. CVDs are a significant public health problem and are an important contributor to the cost of medical care (2). In 2017, 17.8 million people died of CVDs, representing one-third of all death across the globe (1). Moreover, 330 million years of life were lost and 35.6 million years lived with disability in 2017 worldwide (3, 4). In 2030, the projected CVDs mortality would be more than 23 million deaths around the world (5). Both developing and developed countries have experienced higher rates of CVDs mortality in the past decades. However, CVDs were more incident in developed countries (6). Based on the World Health Organization (WHO) report, low- and middle-income countries had over three-quarters of CVDs mortality, which is considered a growing epidemic problem in recent years (7).

The huge burden of CVDs could be attributed to several factors including a sedentary lifestyle, obesity, hypertension, diabetes, excessive alcohol consumption, and unhealthy diet or dietary risk factors (8). Based on GBD 2019 study, the dietary risk factors that can cause CVDs are either an over-consumed diet (sodium, trans-fatty acids, sugar-sweetened beverages, red meat, and processed meat) or an under-consumed diet (whole grains, legumes, vegetables, fruits, nuts and seeds, milk, fiber, calcium, omega-3 fatty acids from seafood, and polyunsaturated fatty acids) (2). Several studies observed the trend of CVDs associated with various risk factors in different regions of the world. For example, Wang et al. (9) reported the trend of IHD attributed to several risk factors (i.e., smoking, low physical activity, air pollution, and dietary risk factors) in the high socio-demographic index (SDI) and low SDI countries from 1990 to 2017. Amini et al. (10) determined the trend of CVDs mortality, incidence, and mortality-to-incidence ratio in different countries based on the Human Development Index (HDI) from 1990 to 2017.

Roth et al. (2) observed the trend of CVDs mortality and DALYs associated with various risk factors ranging from low physical activity, tobacco use, and air pollution, to dietary risk factors at the global, regional, and national levels from 1990 to 2019. Moreover, Wu et al. (11) determined the trend of CVDs mortality (i.e., IHD and stroke) attributed to tobacco exposure in China, Japan, the USA, and the world from 1990 to 2017. However, no or limited studies (2, 9, 12) observed the trend of CVDs (i.e., IHD and IS) mortality and DALYs attributed to modifiable dietary risk factors in the East Asian countries and the world. To advise health policymakers and set standards for decision-makers, accurate, consistent, and comparable analysis of long-term trends and patterns at a country and global level is required. Therefore, we aimed to determine the trend of CVDs mortality and DALYs attributed to modifiable dietary risk factors in East Asian countries and the world (1990–2019) and its prediction from 2020 to 2030.

## Materials and methods

### Data source

In this study, the data were extracted according to sex (male, female, and both sex combined) on ASMR of CVDs and DALYs rates (per 100,000 persons) from the GBD free online database (GBD (13) http://ghdx.healthdata.org/gbd-results-tool) from 1990 to 2019. In addition, according to sex (male and female), age-specific data on CVD mortality and DALYs rates were extracted for different age groups (i.e., 50–54, 55–59, 60–64, 65–69, 70–74, and 75–79 years). GBD is an international cooperative project that estimates the disease burden at regional, national, and global levels. The GBD data is managed by the Institute for Health Metrics and Evaluation (IHME) and analyzed by a group of more than 1,800 researchers in more than 100 countries. GDB estimates the burden of disease indices, including prevalence, incidence, mortality rate, years of life lost (YLL), years lived with disability (YLD), and DALYs for several diseases and injuries. Moreover, the GBD data are provided by different organizations like World Bank Open Data, WHO, and Global Health Observatory for different political and social research (3, 4).

### Variables under study

In the present study, the considered risk factor was a dietary risk factor, and the outcomes were ASMR of IHD and IS and DALYs rates for the East Asian countries and the world from 1990 to 2019. The dietary risk factor was a composite of an over-consumed diet (sodium, trans-fatty acids, sugar-sweetened beverages, red meat, and processed meat) and an under-consumed diet (whole grains, legumes, vegetables, fruits, nuts and seeds, milk, fiber, calcium, omega-3 fatty acids from seafood, and polyunsaturated fatty acids) (2). DALYs are defined as the sum of years lived with disability (YLDs) and years of life lost (YLLs) (4).

### Statistical analysis

#### Joinpoint regression for trend analysis (1990–2019)

To assess the temporal trends of IHD and IS burden, we estimated the average annual percentage change (AAPC) for IHD and IS mortality and DALYs with the joinpoint regression analysis. AAPC represents the trend of IHD and IS burden in the whole period from 1990 to 2019. Additionally, AAPC is a weighted average of the yearly percentage change determined by the joinpoint model, with weights corresponding to the duration of the annual percentage change (APC) interval. The APC shows the IHD and IS burden trend in each segment determined by using the joinpoint regression software. From 1990 to 2019, we produced AAPCs and their 95% confidence intervals (CIs) for each trend segment identified by the model. Furthermore, we estimated AAPCs for each decade (i.e., 1990–1999, 2000–2009, and 2010–2019). Based on age groups (i.e., 50–54, 55–59, 60–64, 65–69, 70–74, and 75–79 years), AAPC for IHD and IS burden was obtained for both males and females from 1990 to 2019. AAPC is considered significant when it is different from 0 at the alpha of 0.05. This analysis was conducted using the joinpoint regression program version 4.8.0.1 (April 2020) from the Surveillance Research Program of the U.S. National Cancer Institute (NCI).

#### ARIMA model for forecasting (2020–2030)

To forecast future CVD mortality and DALY rates, the autoregressive integrated moving average (ARIMA) (p, d, q) model was utilized. It generates forecasts utilizing the shift and lag of historical data, based on past values in the time series (an autoregressive: AR term) and the error caused by previous predictions (a moving average: MA term). In the ARIMA model, integrated (I term) denotes the differencing of raw observed data to keep the time series stationary, i.e., data values are replaced by the difference from prior values. The letters p, d, and q in the ARIMA model stand for the order of autoregression, degree of difference, and order of moving average, respectively. The zero value of any letter (p, d, q) indicates that a particular component is not involved in the model. For instance, ARIMA (2, 1, 0) indicates two AR terms, one degree of difference, and no MA term in the model. To confirm the AR and MA parameters, we constructed the model and determined the autocorrelation function (ACF) and partial autocorrelation function (PACF) of model residuals. To evaluate the best fitting model for data, different goodness-of-fit indices including the lowest value in Bayesian information criterion (BIC) and highest *R*^2^ (the coefficient of determination), ACF, and PACF of residuals were determined (14, 15). We applied the model to predict the CVDs mortality and DALYs rate from 2020 to 2030. Finally, we used the predicted rates and conducted the joinpoint point regression analysis to determine the trend from 2020 to 2030. The analysis was conducted using the SPSS Amos for Windows version 22 (IBM Corporation, Chicago, USA) and the joinpoint regression program version 4.8.0.1 (April 2020) from the Surveillance Research Program of the U.S. National Cancer Institute (NCI).

## Results

### The temporal trend in the ASMR of IHD and IS attributed to dietary risk factors

**Figure 1:**
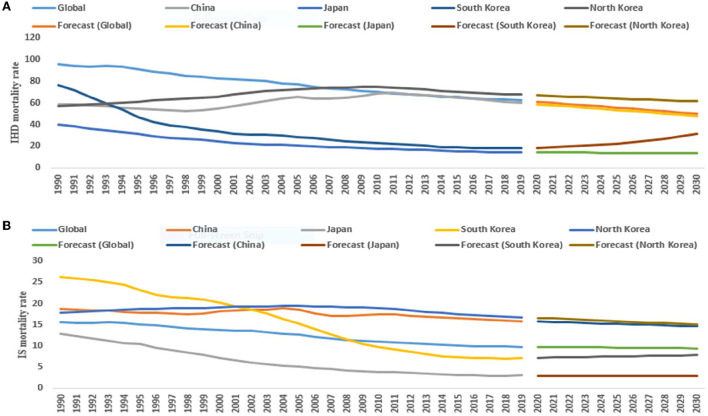
The temporal trends in the ASMR (per 100,000 persons) of IHD (ischemic heart diseases) **(A)** and IS (ischemic stroke) **(B)** (1990–2019) and its prediction (2020–2030) for both sexes in the East Asian countries and world.

For both sexes (1990–2019), the ASMR of IHD attributed to modifiable dietary risk factors in Japan, South Korea, and the world significantly decreased by 3.4% (95% CI: −3.6, −3.3), 4.9% (95% CI: −5.2, −4.7) and 1.4% (95% CI: −1.6, −1.3) per year, respectively. However, the ASMR of IHD in North Korea significantly increased by 0.6% (95% CI: 0.5, 0.8). Moreover, the ASMR of IS in China, Japan, South Korea, North Korea, and the world significantly decreased by 0.6% (95% CI: −0.9, −0.2), 5.0% (95% CI: −5.4, −4.7), 4.5% (95% CI: −4.7, −4.3), 0.2% (95% CI: −0.3, −0.2), and 1.7% (95% CI: −1.8, −1.5) per year, respectively. From 2020 to 2030, the ASMR of IHD and IS attributed to modifiable dietary risk continued to significantly decline in China, North Korea, and the world (Table 1 and Figure 1).

**Table 1:** The temporal trend in the ASMR of IHD and IS attributed to modifiable dietary risk factors in China, Japan, South Korea, North Korea, and the world (1990–2019) and its prediction (2020–2030).

**Trend**		**World**		**China**		**Japan**		**South Korea**		**North Korea**	
**1990–2019**	**Mortality**	**AAPC**	**95%CI**	**AAPC**	**95%CI**	**AAPC**	**95%CI**	**AAPC**	**95%CI**	**AAPC**	**95%CI**
IHD	Both sexes	−1.4^*^	−1.6, −1.3	0.1	−0.2, 0.4	−3.4^*^	−3.6, −3.3	−4.9^*^	−5.2, −4.7	0.6^*^	0.5, 0.8
	Male	−1.4^*^	−1.5, −1.3	0.4^*^	0.2, 0.6	−3.2^*^	−3.4, −3.0	−5.3^*^	−5.7, −4.9	0.5^*^	0.3, 0.6
	Female	−1.6^*^	−1.7, −1.5	−0.2	−0.5, 0.2	−4.1^*^	−4.2, −3.9	−5.0^*^	−5.3, −4.6	0.6^*^	0.5, 0.8
IS	Both sexes	−1.7^*^	−1.8, −1.5	−0.6^*^	−0.9, −0.2	−5.0^*^	−5.4, −4.7	−4.5^*^	−4.7, −4.3	−0.2^*^	−0.3, −0.2
	Male	−1.4^*^	−1.7, −1.1	−0.1	−0.5, 0.3	−4.8^*^	−5.1, −4.5	−4.8^*^	−5.0, −4.5	−0.2^*^	−0.2, −0.1
	Female	−2.0^*^	−2.2, −1.8	−1.2^*^	−1.6, −0.8	−5.5^*^	−6.0, −5.0	−4.4^*^	−4.7, −4.2	−0.4^*^	−0.4, −0.3
**Prediction**											
**2020–2030**											
IHD	Both sexes	−1.9^*^	−2.2, −1.6	−2.0^*^	−2.5, −1.5	−1.3^*^	−1.6, −1.1	4.7^*^	3.4, 6.1	−1.0^*^	−1.0, −0.9
	Male	−1.8^*^	−2.0, −1.6	−2.3^*^	−2.8, −1.8	0.8^*^	0.2, 1.4	−1.9^*^	−2.2, −1.6	−0.8^*^	−0.9, −0.8
	Female	−2.1^*^	−2.5, −1.7	−2.1^*^	−2.6, −1.6	0.2	−0.2, 0.7	0.8^*^	0.1, 1.5	−1.3^*^	−1.4, −1.3
IS	Both sexes	−0.4^*^	−0.7, −0.1	−0.9^*^	−0.1, −0.7	−0.3	−0.7, 0.1	1.0	−0.3, 2.3	−1.1^*^	−1.1, −0.9
	Male	−1.5^*^	−1.6, −1.5	−0.5^*^	−0.7, −0.3	2.1^*^	1.5, 2.7	−0.1	−0.9, 0.7	−1.1^*^	−1.1, -1, 0
	Female	−0.2	−0.6, 0.2	−1.3^*^	−1.5, −1.1	0.1	−0.3, 0.5	1.1	−0.5, 2.7	−1.4^*^	−1.4, −1.3

IHD, Ischemic heart disease; IS, ischemic stroke; AAPC, average annual percent change,

* statistically significant (*p* < 0.05).

**Figure 2:**
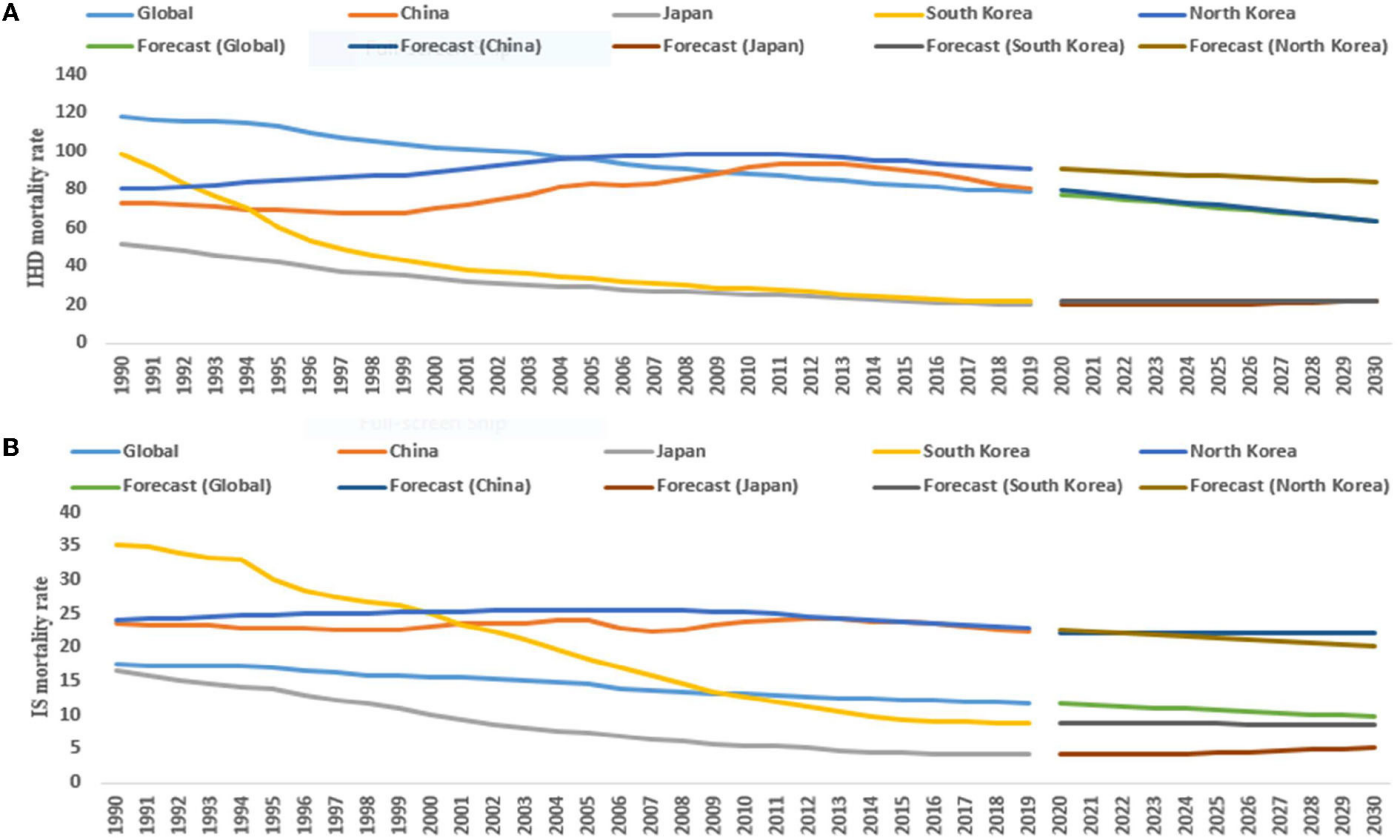
The temporal trends in the ASMR (per 100,000 persons) of IHD (ischemic heart diseases) **(A)** and IS (ischemic stroke) **(B)** (1990–2019) and its prediction (2020–2030) for males in the East Asian countries and world.

For male subjects (1990–2019), the ASMR of IHD significantly decreased by 3.2% (95% CI: −3.4, −3.0) in Japan, 5.3% (95% CI: −5.7, −4.9) in South Korea, and 1.4% (95% CI: −1.5, −1.3) per year in the world. On the other hand, the ASMR of IHD significantly increased by 0.4% (95% CI: 0.2, 0.6) and 0.5% (95% CI: 0.3, 0.6) per year in China and North Korea, respectively. From 2020 to 2030, the ASMR of IHD and IS significantly decreased in China, North Korea, and the world (Table 1 and Figure 2).

**Figure 3:**
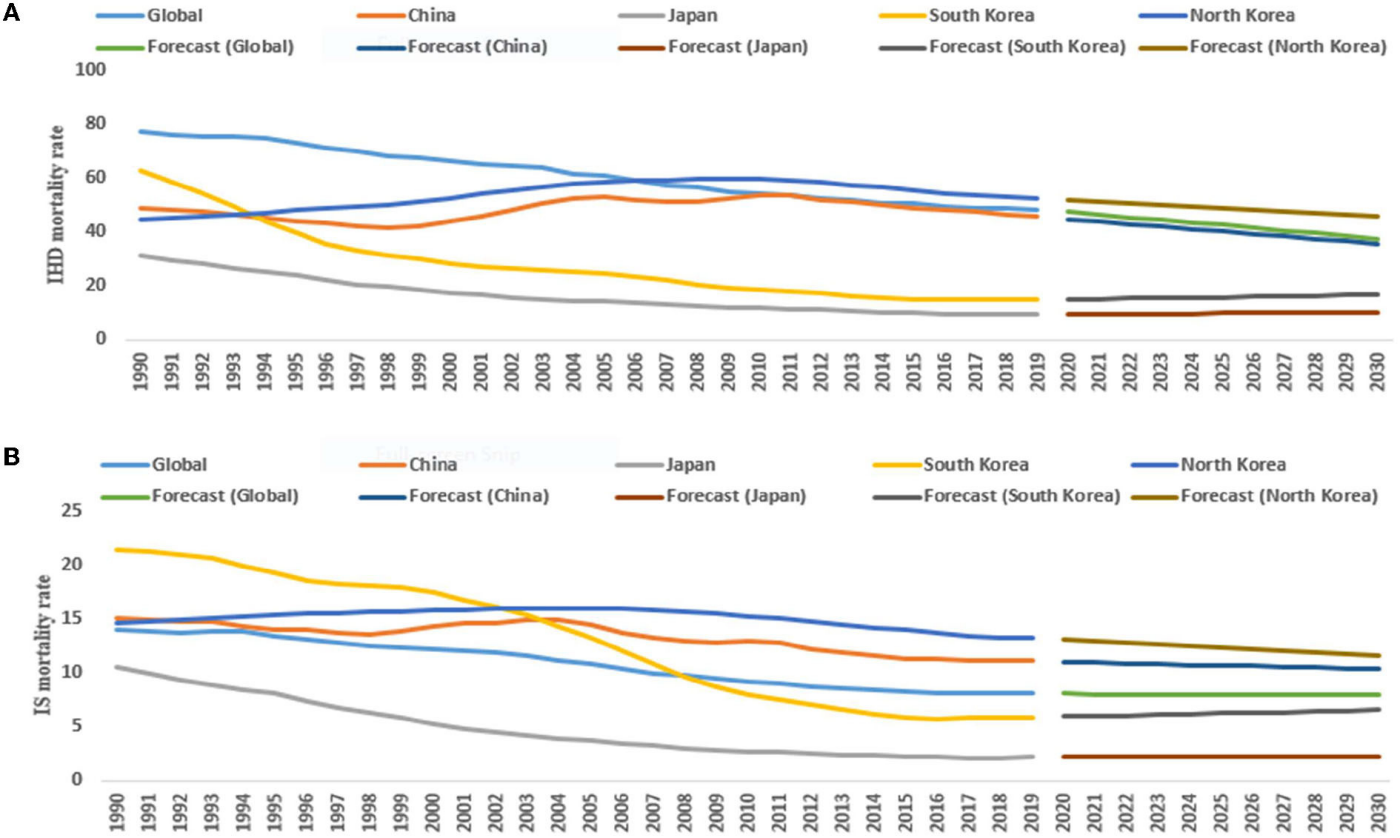
The temporal trends in the ASMR (per 100,000 persons) of IHD (ischemic heart diseases) **(A)** and IS (ischemic stroke) **(B)** (1990–2019) and its prediction (2020–2030) for females in the East Asian countries and world.

For female subjects (1990–2019), the ASMR of IHD in Japan, South Korea, and the world significantly decreased by 4.1% (95% CI: −4.2, −3.9), 5.0% (95% CI: −5.3, −4.6) and 1.6% (95% CI: −1.7, −1.5) per year, respectively, but significantly increased in North Korea by 0.6% (95% CI: 0.5, 0.8). From 2020 to 2030, the ASMR of IHD and IS significantly decreased in China and North Korea (Table 1 and Figure 3).

### The temporal trend in the age-standardized DALYs rate of IHD and IS attributed to dietary risk factors

For both sexes (1990–2019), the age-standardized DALYs rate of IHD attributed to modifiable dietary risk factor significantly decreased by 0.4% (95% CI: −0.6, −0.1), 3.0% (95% CI: −3.2, −2.8), 5.3% (95% CI: −5.5, −5.2), and 1.3% (95% CI: −1.4, −1.2) per year in China, Japan, South Korea, and the world, respectively. However, the age-standardized DALYs rate of IHD significantly increased by 0.5% (95% CI: 0.4, 0.6) per year in North Korea. Furthermore, the age-standardized DALYs rate of IS in China, Japan, South Korea, North Korea, and the world significantly decreased by 0.5% (95% CI: −1.0, −0.1), 3.7% (95% CI: −3.9, −3.5), 4.3% (95% CI: −4.5, −4.1), 0.2% (95% CI: −0.3, −0.2), and 1.4% (95% CI: −1.5, −1.2) per year, respectively. From 2020 to 2030, the age-standardized DALYs rate of IHD and IS attributed to dietary risk significantly declined in China, North Korea, and the world but significantly increased in Japan and South Korea (Table 2 and Supplementary Figure S1).

**Table 2:** The temporal trend in the age-standardized DALYs rate of IHD and IS attributed to modifiable dietary risk factors in China, Japan, South Korea, North Korea, and the world (1990–2019) and its prediction (2020–2030).

**Trend**		**World**		**China**		**Japan**		**South Korea**		**North Korea**	
**1990-2019**	**DALYs**	**AAPC**	**95%CI**	**AAPC**	**95%CI**	**AAPC**	**95%CI**	**AAPC**	**95%CI**	**AAPC**	**95%CI**
IHD	Both sexes	−1.3^*^	−1.4, −1.2	−0.4^*^	−0.6, −0.1	−3.0^*^	−3.2, −2.8	−5.3^*^	−5.5, −5.2	0.5^*^	0.4, 0.6
	Male	−1.3^*^	−1.5, −1.0	−0.0	−0.2, 0.2	−2.8^*^	−3.0, −2.7	−5.5^*^	−5.9, −5.1	0.4^*^	0.3, 0.5
	Female	−1.5^*^	−1.6, −1.3	−0.8^*^	−1.0, −0.6	−4.0^*^	−4.1, −3.8	−5.7^*^	−5.9, −5.5	0.5^*^	0.4, 0.6
IS	Both sexes	−1.4^*^	−1.5, −1.2	−0.5^*^	−1.0, −0.1	−3.7^*^	−3.9, −3.5	−4.3^*^	−4.5, −4.1	−0.2^*^	−0.3, −0.2
	Male	−1.2^*^	−1.5, −0.9	−0.2	−0.5, 0.1	−3.8^*^	−4.0, −3.6	−4.5^*^	−4.8, −4.3	−0.2^*^	−0.3, −0.2
	Female	−1.6^*^	−1.7, −1.4	−1.0^*^	−1.4, −0.6	−3.8^*^	−4.1, −3.6	−4.1^*^	−4.4, −3.9	−0.3^*^	−0.4, −0.3
**Prediction**											
**2020–2030**											
IHD	Both sexes	−1.5^*^	−1.6, −1.5	−2.0^*^	−2.5, −1.6	0.8^*^	0.3, 1.4	6.0^*^	4.5, 7.4	−0.8^*^	−0.8.−0.7
	Male	−1.4^*^	−1.5, −1.4	−2.0^*^	−2.5, −1.5	−0.2	−0.7, 0.3	−2.0^*^	−2.3, −1.6	−0.8^*^	−0.8.−0.7
	Female	−1.7^*^	−1.7, −1.6	−2.0^*^	−2.3, −1.6	−0.2	−0.6, 0.2	6.8^*^	5.5, 8.0	−1.2^*^	−1.2, −1.1
IS	Both sexes	−0.8^*^	−1.0, −0.6	−0.5^*^	−0.6, −0.4	1.4^*^	1.0, 1.9	1.2^*^	0.2, 2.2	−0.9^*^	−0.9, −0.8
	Male	−0.6^*^	−0.8, −0.4	−0.1^*^	−0.1, −0.0	−0.7^*^	−1.1, −0.4	−0.3	−1.0, 0.4	−1.0^*^	−1.0, −0.9
	Female	−0.1	−0.4, 0.1	−0.4	−0.9, 0.1	2.0^*^	1.6, 2.4	1.8^*^	0.6, 3.1	−1.0^*^	−1.0, −0.9

DALYs, Disability-adjusted life years, IHD, Ischemic heart disease; IS, ischemic stroke; AAPC, average annual percent change,

* statistically significant (*p* < 0.05).

The age-standardized DALYs rate of IHD in the male population (1990–2019) significantly decreased by 2.8% (95% CI: −3.0, −2.7) in Japan, 5.5% (95% CI: −5.9, −5.1) in South Korea, and 1.3% (95% CI: −1.5, −1.0) per year in the world. However, the age-standardized DALYs rate of IHD significantly increased by 0.4% (95% CI: 0.3, 0.5) per year in North Korea. From 2020 to 2030, the age-standardized DALYs rate of IHD and IS significantly decreased in China, North Korea, and the world (Table 2 and Supplementary Figure S2).

In female population (1990–2019), the age-standardized DALYs rate of IHD in China, Japan, South Korea and the world significantly decreased by 0.8% (95% CI: −1.0, −0.6), 4.0% (95% CI: −4.1, −3.8), 5.7% (95% CI: −5.9, −5.5), and 1.5% (95% CI: −1.6, −1.3) per year, respectively, but significantly increased by 0.5% (95% CI: 0.4, 0.6) in North Korea. From 2020 to 2030, the age-standardized DALYs rate of IHD and IS significantly declined in North Korea but significantly increased in South Korea (Table 2 and Supplementary Figure S3).

### The ASMR and DALYs rate of IHD and IS in the East Asian countries and the world in different decades

Regardless of the sex (male, female, and both sex combined), a significant downward trend in the ASMR and DALYs rate of IHD and IS was observed in all three decades (i.e., 1990–1999, 2000–2009, and 2010–2019) in Japan, South Korea, and the world. However, the ASMR and DALYs rate of IHD significantly increased in China (2000–2009) and North Korea (1990–1999 and 2000–2009) (Supplementary Tables S1–S6).

### The ASMR and DALYs rate of IHD and IS by age groups

Both in males and females, the mortality and DALYs rate of IHD and IS significantly declined in all age groups in Japan, South Korea, and the world from 1990 to 2019. Moreover, the declining trend of IHD and IS mortality and DALYs rates was higher in females than those in males regardless of the age groups. However, the mortality and DALYs rate of IHD showed a significant upward trend in the male aged group (75–79 years) in China. In North Korea, the mortality and DALYs rate of IHD significantly increased in all age groups regardless of sex (Supplementary Tables S7–S12).

### Ranking of dietary risk factors attributed to IHD and IS

**Figure 4:**
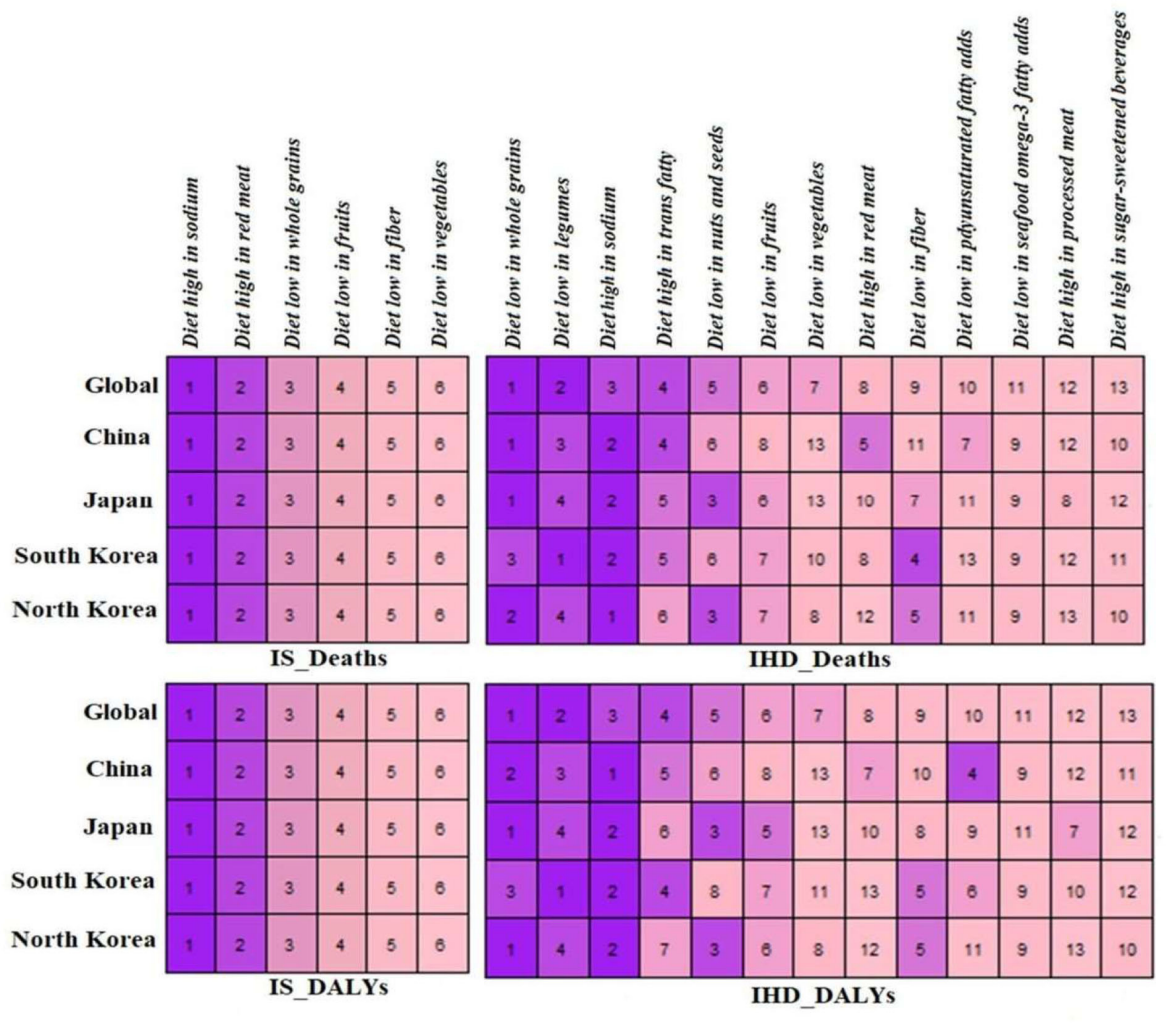
Ranking the dietary risk factors for mortality and DALYs due to ischemic heart disease (IHD), ischemic stroke (IS) for world, China, Japan, South Korea and North Korea, 2019.

Figure 4 shows the ranking of dietary risk factors attributed to the mortality and DALYs of IHD and IS in the East Asian countries and the world in 2019. Globally, a diet low in whole grains was the most significant factor in IHD mortality (18.6 per 100,000 persons) and DALYs (393.5 per 100,000 persons) in 2019, followed by a diet low in legumes and a diet high in sodium (Supplementary Tables S13, S14). Dietary risk factors including a diet low in whole grains (China and Japan), a diet high in sodium (North Korea), and a diet low in legumes (South Korea) were the leading risk factors of IHD mortality in the East Asian countries (Supplementary Table S13). Moreover, a diet high in sodium, a diet high in red meat, and a diet low in whole grains were the leading risk factors for higher IS mortality and DALY the East Asia countries and the world (Supplementary Table S15).

## Discussion

The study of the GBD 2019 data revealed the trend of ASMR of IHD and IS and DALYs rate attributed to modifiable dietary risk factors in the East Asian countries and the world (1990–2019), and its prediction from 2020 to 2030. We observed that regardless of the sex (male, female, and both sex combined), the ASMR and DALYs rate of IHD attributed to modifiable dietary risk factors significantly decreased in Japan, South Korea, and the world. However, the ASMR of IHD significantly increased in China (male) and North Korea (male, female, and both sex combined) from 1990 to 2019. Moreover, the ASMR and DALYs rate of IHD and IS significantly decreased in China, North Korea, and the world (male and both sex combined) from 2020 to 2030. Globally, a diet low in whole grains was the most significant factor in IHD mortality and DALYs in 2019, followed by a diet low in legumes and a diet high in sodium. A diet low in whole grains (China and Japan), a diet high in sodium (North Korea), and a diet low in legumes (South Korea) were the leading risk factors of IHD mortalin the East Asian countries.

### The temporal trend in IHD and IS burden in the East Asian countries and the world

IHD and IS are the primary consequences of dietary risk factors. These dietary risk factors are the sum of either an over-consumed diet (sodium, trans-fatty acids, sugar-sweetened beverages, red meat, and processed meat) or an under-consumed diet (whole grains, legumes, vegetables, fruits, nuts and seeds, milk, fiber, calcium, omega-3 fatty acids from seafood, and polyunsaturated fatty acids). Globally, 7.94 million CVD annual deaths and 188 million annual DALYs are attributed to dietary risk factors (2). Based on 2017 data, 10 million CVD mortality and 207 million DALYs were due to dietary risk factors (16).

Our study found that for both sexes, the ASMR of IHD due to dietary risk factors significantly decreased (AAPC; −1.4%) from 95.6 to 62.4 per 100,000 persons from 1990 to 2019. The age-standardized DALYs rate of IHD significantly decreased (AAPC; −1.3%) from 1876.6 in 1990 to 1271.3 per 100,000 persons in 2019 in the world. Similarly, Dong et al. (12) reported a significant decrease in the age-standardized diet-related overall CVDs mortality (138.5–87.4 per 100,000 persons) and DALYs rate (2880.6–1870.1 per 100,000 persons) with an overall estimated annual percentage change (EAPC) of −1.3% from 1990 to 2019.

Our study also observed that the ASMR and DALYs rate of IHD and IS due to dietary risk factors were higher in male subjects than in female subjects. Moreover, the negative AAPC in the ASMR and DALYs rate of IHD and IS are comparatively lower in males than in the female population in the world, suggesting a milder declining trend in the mortality and DALYs rate of IHD and IS in males. Dong et al. (12) also observed that due to dietary risk factors, men suffered higher CVDs deaths and DALYs burden than women worldwide. These differences reflect the distribution of different risk factors between genders. Furthermore, several pathophysiological factors, the protective role of estrogen, and differences in vascular hemodynamics may also play some significant roles in gender differences (17–19).

Among the East Asian countries (for both sexes), Japan and South Korea experienced a significantly decreasing trend in the ASMR and DALYs rate of IHD and IS, which could be linked to their healthy food patterns. The Japanese diet primarily consists of seafood, fruits, and vegetables (20). According to a Japanese cohort study, adults who followed the Japanese dietary guidelines at baseline had lower rates of CVD deaths than those who did not (21). In a GBD study of cardiovascular diseases and risk factors (1990–2019), Roth et al. (2) reported a decreasing trend of age-standardized DALYs rate due to IHD in Japan and South Korea. Healthy diet habits improved living standards, and diagnosis of CVDs at an earlier stage could be attributed to the decreases in the ASMR and DALYs rate of IHD and IS (22, 23). However, in a national-level cross-sectional survey (1998–2016), the IHD deaths due to dietary risk factors substantially increased in South Korea during the study period (24).

Due to the dietary risk factors, the trend of ASMR and DALYs rate of IHD significantly increased in North Korea (male, female, and both sex combined). North Korea had about four-fold higher ASMR and DALYs rate of IHD compared with South Korea. In a GBD study in 2004, WHO estimated that North Korea had three times higher CVD than South Korea (25). In the South-East Asian Region (SEAR), North Korea had the highest death rate from CVD (26). The higher rates of CVD mortality in the North Korean population could be attributed to their exposure to malnutrition in their infancy and developmental phase during the time of economic hardship (mid-to-late-1990s) (27).

In China, males experienced two times higher ASMR of IHD compared with females and had a significantly increased trend of IHD mortality from 1990 to 2019, which is comparable with the GBD study (28). Based on previous reports, the incidence of coronary heart disease occurs later (10–15 years) in women than in men. Moreover, due to the protective effect of estrogen on the cardiovascular system, women had a lower incidence of CVD than men (29, 30). China observed an increasing trend of diet-related CVD death and DALYs rates (12). The “Healthy China Action Plan (2019–2030)”, the most recent health and nutrition-related policy has been launched including the popularization of health knowledge, promotion of a balanced diet, and physical activities to reduce CVD mortality and morbidity (31). Moreover, the differences in the ASMR and DALYs rate of IHD and IS among the East Asian countries could be due to variations in diet quality, lack of knowledge related to a healthy diet, and levels of exposure to different dietary risk factors (2).

The ASMR of IHD and IS due to dietary risk factors tended to decline until 2030 in China, North Korea (male, female, and both sex combined), and the world (male and both sex combined). However, a significant upward trend was observed in Japanese male subjects. In addition, the age-standardized DALYs rate of IHD and IS continued to decline until 2030 in China, North Korea, and the world (male and both sex combined) but significantly increased in Japan (both sexes) and South Korea (female and both sexes). In Japan, high salt intake is considered a national epidemic (32, 33), and the predicted increasing trend of IHD and ischemic burden could be attributed to high salt consumption. In our findings, a diet high in sodium is the leading risk factor for high IS mortality and DALYs and the second risk factor for high IHD mortality and DALYs in Japan. The latest National Health and Nutrition Survey (NHNS) in 2016 reported that the average daily salt consumption in the Japanese population was 9.9 g (10.8 g for men and 9.1 g for women) (34) which is much larger than the WHO's recommended level (<5.0 g per day) (35).

### The IHD and IS burden in different decades

We observed a significant downward trend in the ASMR and DALYs rate of IHD and IS attributed to dietary risk factors in all three decades in Japan, South Korea, and the world. However, the ASMR and DALYs rate of IHD significantly increased in China from 2000to 2009 and in North Korea from 1990 to 1999 and 2000 to 2009. Tian et al. (28) reported an increased trend of IHD DALYs attributed to dietary risk factors in the Chinese population from 1999 to 2010, which is in accordance with our findings. Diet is one of the key risk factors among the many established risk factors associated with the CVD burden (3). Several complex factors influence diet quality and food choices such as the economy, culture, and the nutrition environment (36).

In China, the rapid economic development and cultural exchanges have shifted people's food consumption choices from a traditional diet to a westernized diet which may contribute to observed increases in chronic diseases. A westernized diet such as the fast-food (FF) industry has increased rapidly in China in the last two decades (37, 38). The number of people who eat FF has increased by 40.20% from 14.70% in 2000 to 20.61% in 2008 in China (39). Moreover, the total revenue (in million US$) of FF increased from 10,464 to 94,218 from 1999to 2013, indicating an increased trend in FF consumption in China (40). Fast-food consumption (FFC) can increase the risk of CVD (41) and the rapid FFC could be one of the attributable risk factors for the high burden of IHD deaths and DALYs from 1999 to 2010 in China.

### The IHD and IS burden in different age groups

Regardless of sex, the IHD and IS burden attributed to dietary risk factors significantly declined in all age groups in Japan, South Korea, and the world. However, the mortality and DALYs rate of IHD significantly increased in all age groups in North Korea. The transition in diet from traditional Asian diets to westernized diets has been observed in several Asian countries and is attributable to the high CVDs burden in the region (42). A review article reported increasing consumption of animal source food, oil, sugar, and sugar-sweetened beverages, and low consumption of whole grains and legumes in low- to middle-income countries (43). In China, the mortality and DALYs rate of IHD showed a significant upward trend in the male aged group (75–79 years). Tian et al. (28) also reported that the DALYs rate of IHD due to dietary risk factors was higher in Chinese people over 80 years old. The risk CVDs mortality and morbidity increases with increasing age (44) and could be attributed to an alteration in the arteries, atherosclerosis, decreased elasticity, and fibrosis (45).

### Ranking of dietary risk factors

In 2019, a diet low in whole grains was the most significant risk factor for IHD mortality and DALYs, followed by a diet low in legumes, and a diet high in sodium in the world. Diet groups including a diet low in whole grains (China and Japan), a diet high in sodium (North Korea), and a diet low in legumes (South Korea) were the leading risk factors of IHD mortality in the East Asian countries. Moreover, a diet high in sodium, a diet high in red meat, and a diet low in whole grains were the leading risk factors for higher IS mortality and DALYs rate in East Asia countries and the world. It has been observed that two-thirds of CHD and two-fifth of acute ischemic stroke events could be avoided by adopting a healthy lifestyle (46, 47). A balanced diet is one of the significant parts of a healthy lifestyle (48). An unhealthy diet is associated with a high burden of CVD in Asian countries and the world (16, 42).

In a GBD study in 2017, CVD was the leading cause of deaths (10 million) and DALYs (207 million) attributed to dietary risk factors. A diet low in whole grains (3 million deaths and 82 million DALYs), a diet high in sodium (3 million deaths and 70 million DALYs), and a diet low in fruits (2 million deaths and 65 million DALYs) were the leading dietary risk factors for overall deaths and DALYs globally and in many counties (16). In a recent GBD study in 2019, around 6.9 million CVD deaths and 153.2 million DALYs are attributed to dietary risk factors. A diet low in whole grains significantly contributed to higher CVD deaths DALYs followed by a high intake of sodium, a diet low in fruits, a diet low in vegetables, and a diet low in seafood omega-3 fatty acids which are comparable with our findings (12). High intake of sodium is the leading dietary risk factor for IHD DALYs, IS deaths, and DALYs, and the second risk factor for IHD deaths in the Chinese population. In China, the salt intake is higher (12 g/person/day) than the WHO recommended level (5 g/person/day), which is associated with a higher prevalence of hypertension (49).

## Limitations

Although IHME provides standard and improved high-quality estimates of the global burden of diseases still we had certain limitations in this study. First, our analysis relies on the GBD secondary data and all GBD limitations are also applicable to our findings as described previously (16, 50). Second, GBD does not provide sub-types of IHD such as non-ST-segment elevation myocardial infarction (NSTEMI), unstable angina (UA), or ST-segment elevation myocardial infarction (STEMI). Third, our analyses are limited to people aged ≥50 years. Finally, out of fifteen dietary risk factors (16), 13 dietary risk factors for IHD deaths and DALYs and only six dietary risk factors were available for IS deaths and DALYs estimates in the East Asian countries and the world. Moreover, we have described trends of IHD and IS burden attributed to overall dietary risk factors and could not find trends for the individual dietary risk factor.

## Conclusion

This study found that regardless of the sex, ASMR, and DALYs rate of IHD attributed to modifiable dietary risk factors significantly decreased in Japan, South Korea, and the world. Globally, the ASMR and DALYs rate of IHD and IS due to dietary risk factors were higher in male subjects than in female subjects. Among East Asian countries, the decreasing trend of IHD and IS burden in South Korea is remarkable compared with the world. However, the ASMR of IHD significantly increased in China (male) and North Korea (male, female, and both sex combined) from 1990 to 2019. From 2020 to 2030, the ASMR of IHD is predicted to increase in South Korea (female) and Japan (male). Globally, a diet low in whole grains was the top risk factor for highest IHD mortality and DALYs in 2019, followed by a diet low in legumes and a diet high in sodium. Diet low in whole grains, a diet high in sodium, and a diet low in legumes were the leading risk factors of high IHD mortality in East Asian countries. These estimates may facilitate the public health policymakers across these countries to take appropriate measures and design proper strategies to further overcome the CVDs burden attributed to dietary risk factors.

## Data availability statement

The original contributions presented in the study are included in the article/Supplementary material, further inquiries can be directed to the corresponding author.

## Ethics statement

Ethical review and approval was not required for the study on human participants in accordance with the local legislation and institutional requirements. Written informed consent for participation was not required for this study in accordance with the national legislation and the institutional requirements.

## Author contributions

N: conceptualization, data curation, formal analysis, methodology, software, validation, visualization, writing—original draft, and writing—review and editing. WB: validation, visualization, and writing—review and editing. ZL: data curation, software, validation, visualization, and investigation. SM: data curation, validation, visualization, investigation, and writing—review. GF and YW: investigation, resources, validation, funding acquisition, project administration, and supervision. All authors contributed to the article and approved the submitted version.

## Funding

This work is supported by the feasibility study of the ecological follow-up model for a regional collaborative coronary intervention project (Grant No: 2019YFE0113900).

## Conflict of interest

The authors declare that the research was conducted in the absence of any commercial or financial relationships that could be construed as a potential conflict of interest.

## Publisher's note

All claims expressed in this article are solely those of the authors and do not necessarily represent those of their affiliated organizations, or those of the publisher, the editors and the reviewers. Any product that may be evaluated in this article, or claim that may be made by its manufacturer, is not guaranteed or endorsed by the publisher.
