# Supplementary material for: Prediction of cardiovascular diseases mortality- and disability-adjusted life-years attributed to modifiable dietary risk factors from 1990 to 2030 among East Asian countries and the world
Source: Front Nutr. 2022 Oct 17;9:898978. doi: 10.3389/fnut.2022.898978 (PMC9618868; doi:10.3389/fnut.2022.898978)
Supplement: Supplementary file 1 [file Data_Sheet_1.docx]

**Table S1:** The temporal trend of the ASMR of IHD and IS attributed to modifiable dietary risk factor in both sexes population of China, Japan, South Korea, North Korea, and the world (1990-1999, 2000-2009, 2010-2019)

| Mortality |  | World | | China | | Japan | | South Korea | | North Korea | |
| --- | --- | --- | --- | --- | --- | --- | --- | --- | --- | --- | --- |
| Both sexes | Trends | AAPC | 95%CI | AAPC | 95%CI | AAPC | 95%CI | AAPC | 95%CI | AAPC | 95%CI |
| IHD | 1990-1999 | -1.3^*^ | -1.6,-1.0 | -0.9^*^ | -1.5,-0.3 | -4.9^*^ | -5.1,-4.7 | -8.4^*^ | -8.9,-8.0 | 1.7^*^ | 1.6,1.8 |
|  | 2000-2009 | -1.7^*^ | -1.7,-1.6 | 2.2^*^ | 1.9,2.6 | -3.1^*^ | -3.2,3.0 | -3.9^*^ | -4.1,-3.8 | 1.3^*^ | 1.0,1.6 |
|  | 2010-2019 | -1.3^*^ | -1.6,-1.1 | -1.6^*^ | -2.1,-1.0 | -2.2^*^ | -2.8,-1.7 | -2.6^*^ | -3.3,-1.9 | -1.2^*^ | -1.3,-1.0 |
| IS | 1990-1999 | -1.3^*^ | -1.5,-1.1 | -0.6^*^ | -1.1,-0.1 | -5.4^*^ | -6.2,-4.7 | -2.8^*^ | -3.1,-2.6 | 0.7^*^ | 0.6,0.8 |
|  | 2000-2009 | -2.3^*^ | -2.5,-2.1 | -0.4 | -0.9,0.1 | -6.6^*^ | -6.9,-6.3 | -6.6^*^ | -6.8,-6.3 | -0.1 | -0.1,0.0 |
|  | 2010-2019 | -1.3^*^ | -1.7,-1.0 | -0.9^*^ | -1.1,-0.8 | -3.1^*^ | -3.6,-2.6 | -3.9^*^ | -4.5,-3.4 | -1.3^*^ | -1.3,-1.2 |

**Note:** Ischemic heart disease (IHD), ischemic stroke (IS), average annual percent change (AAPC), *: statistically significant (p < 0.05).

**Table S2:** The temporal trend of the age-standardized DALYs rate of IHD and IS attributed to modifiable dietary risk factor in both sexes population of China, Japan, South Korea, North Korea, and the world (1990-1999, 2000-2009, 2010-2019)

| DALYs |  | World | | China | | Japan | | South Korea | | North Korea | |
| --- | --- | --- | --- | --- | --- | --- | --- | --- | --- | --- | --- |
| Both sexes | Trends | AAPC | 95%CI | AAPC | 95%CI | AAPC | 95%CI | AAPC | 95%CI | AAPC | 95%CI |
| IHD | 1990-1999 | -1.1^*^ | -1.3,-0.8 | -1.2^*^ | -1.7,-0.7 | -4.2^*^ | -4.4,-4.0 | -9.2^*^ | -9.5,-9.0 | 1.5^*^ | 1.5,1.6 |
|  | 2000-2009 | -1.6^*^ | -1.6,-1.5 | 1.2^*^ | 0.9,1.5 | -2.6^*^ | -2.7,-2.5 | -4.1^*^ | -4.2,-4.0 | 1.0^*^ | 0.8,1.2 |
|  | 2010-2019 | -1.3^*^ | -1.5,-1.0 | -1.4^*^ | -1.9,-0.9 | -2.2^*^ | -2.8,-1.6 | -2.8^*^ | -3.2,-2.4 | -1.0^*^ | -1.1,-0.8 |
| IS | 1990-1999 | -1.1^*^ | -1.3,-1.0 | -0.8^*^ | -1.4,-0.2 | -4.0^*^ | -4.4,-3.5 | -2.7^*^ | -3.0,-2.4 | 0.6^*^ | 0.5,0.6 |
|  | 2000-2009 | -2.0^*^ | -2.3,-1.6 | -0.4 | -0.8,0.1 | -5.2^*^ | -5.5,-4.9 | -6.2^*^ | -6.4,-6.0 | -0.2^*^ | -0.3,-0.2 |
|  | 2010-2019 | -0.9^*^ | -1.2,-0.6 | -0.6^*^ | -0.7,-0.5 | -2.0^*^ | -2.4,-1.7 | -4.0^*^ | -4.4,-3.5 | -1.0^*^ | -1.1,-1.0 |

**Note:** Disability-adjusted life years (DALYs), ischemic heart disease (IHD), ischemic stroke (IS), average annual percent change (AAPC), *: statistically significant (p < 0.05).

**Table S3:** The temporal trend of the ASMR of IHD and IS attributed to modifiable dietary risk factor in the male population of China, Japan, South Korea, North Korea, and the world (1990-1999, 2000-2009, 2010-2019)

| Mortality |  | World | | China | | Japan | | South Korea | | North Korea | |
| --- | --- | --- | --- | --- | --- | --- | --- | --- | --- | --- | --- |
| Male | Trends | AAPC | 95%CI | AAPC | 95%CI | AAPC | 95%CI | AAPC | 95%CI | AAPC | 95%CI |
| IHD | 1990-1999 | -1.4^*^ | -1.8,-0.9 | -0.6^*^ | -1.0,-0.3 | -4.3^*^ | -4.5,-4.1 | -9.0^*^ | -9.8,-8.2 | 1.2^*^ | 1.2,1.3 |
|  | 2000-2009 | -1.4^*^ | -1.5,-1.4 | 2.5^*^ | 2.3,2.8 | -2.8^*^ | -2.9,-2.7 | -3.6^*^ | -4.0,-3.3 | 1.0^*^ | 0.6,1.4 |
|  | 2010-2019 | -1.4^*^ | -1.5,-1.4 | -1.3^*^ | -1.7,-0.9 | -2.4^*^ | -3.0,-1.7 | -3.3^*^ | -3.5,-3.1 | -1.0^*^ | -1.1,-0.8 |
| IS | 1990-1999 | -1.2^*^ | -1.3,-1.1 | -0.0 | -0.2,0.1 | -4.5^*^ | -5.2,-3.9 | -3.7^*^ | -4.0,-3.4 | 0.5^*^ | 0.4,0.6 |
|  | 2000-2009 | -1.9^*^ | -2.7,-1.0 | 0.1 | -0.2,0.5 | -6.3^*^ | -6.7,-5.9 | -6.4^*^ | -6.6,-6.2 | 0.0 | -0.0,0.1 |
|  | 2010-2019 | -1.2^*^ | -1.4,-1.1 | -0.7 | -1.4,0.1 | -3.5^*^ | -4.0,-3.0 | -4.1^*^ | -4.7,-3.5 | -1.1^*^ | -1.1,-1.0 |

**Note:** Ischemic heart disease (IHD), ischemic stroke (IS), average annual percent change (AAPC), *: statistically significant (p < 0.05).

**Table S4:** The temporal trend of the age-standardized DALYs rate of IHD and IS attributed to modifiable dietary risk factor in the male population of China, Japan, South Korea, North Korea, and the world (1990-1999, 2000-2009, 2010-2019)

| DALYs |  | World | | China | | Japan | | South Korea | | North Korea | |
| --- | --- | --- | --- | --- | --- | --- | --- | --- | --- | --- | --- |
| Male | Trends | AAPC | 95%CI | AAPC | 95%CI | AAPC | 95%CI | AAPC | 95%CI | AAPC | 95%CI |
| IHD | 1990-1999 | -1.0^*^ | -1.8,-0.3 | -1.1^*^ | -1.4,-0.7 | -3.6^*^ | -3.8,-3.4 | -9.3^*^ | -10.0,-8.7 | 1.2^*^ | 1.2,1.3 |
|  | 2000-2009 | -1.4^*^ | -1.4,-1.3 | 1.7^*^ | 1.5,1.9 | -2.2^*^ | -2.5,-1.9 | -3.8^*^ | -4.1,-3.5 | 0.8^*^ | 0.6,1.0 |
|  | 2010-2019 | -1.4^*^ | -1.4,-1.3 | -1.0^*^ | -1.4,-0.7 | -2.7^*^ | 3.0,-2.4 | -3.5^*^ | -3.6,-3.3 | -0.9^*^ | -1.0,-0.8 |
| IS | 1990-1999 | -1.0^*^ | -1.1,-0.8 | -0.6^*^ | -1.1,-0.0 | -3.6^*^ | -4.1,-3.1 | -3.4^*^ | -3.6,-3.1 | 0.5^*^ | 0.4,0.5 |
|  | 2000-2009 | -1.6^*^ | -2.6,-0.7 | 0.1 | -0.1,0.3 | -5.2^*^ | -5.5,5.0 | -6.1^*^ | -6.2,-5.9 | -0.2^*^ | -0.3,-0.2 |
|  | 2010-2019 | -1.0^*^ | -1.2,-0.8 | -0.3 | -1.2,0.5 | -2.5^*^ | -2.9,-2.2 | -4.2^*^ | -4.7,-3.6 | -0.9^*^ | -1.0,-0.9 |

**Note:** Disability-adjusted life years (DALYs), ischemic heart disease (IHD), ischemic stroke (IS), average annual percent change (AAPC), *: statistically significant (p < 0.05).

**Table S5:** The temporal trend of the ASMR of IHD and IS attributed to modifiable dietary risk factor in the female population of China, Japan, South Korea, North Korea, and the world (1990-1999, 2000-2009, 2010-2019)

| Mortality |  | World | | China | | Japan | | South Korea | | North Korea | |
| --- | --- | --- | --- | --- | --- | --- | --- | --- | --- | --- | --- |
| Female | Trends | AAPC | 95%CI | AAPC | 95%CI | AAPC | 95%CI | AAPC | 95%CI | AAPC | 95%CI |
| IHD | 1990-1999 | -1.4^*^ | -1.7,-1.1 | -1.5^*^ | -2.1,1.0 | -5.7^*^ | -5.9,-5.5 | -8.0^*^ | -8.6,-7.4 | 1.9^*^ | 1.8,2.0 |
|  | 2000-2009 | -1.9^*^ | -2.0,-1.9 | 1.7^*^ | 1.1,2.3 | -4.0^*^ | -4.1,-3.8 | -4.3^*^ | -4.5,-4.1 | 1.4^*^ | 1.0,1.7 |
|  | 2010-2019 | -1.4^*^ | -1.6,-1.1 | -1.0^*^ | -1.3,-0.7 | -2.4^*^ | -2.9,-1.9 | -2.6^*^ | -3.5,-1.8 | -1.5^*^ | -1.7,-1.3 |
| IS | 1990-1999 | -1.5^*^ | -1.8,-1.3 | -0.9^*^ | -1.5,-0.4 | -7.1^*^ | -7.3,-6.8 | -2.2^*^ | -2.5,-1.9 | 0.8^*^ | 0.6,0.9 |
|  | 2000-2009 | -2.7^*^ | -2.9,-2.5 | -1.0^*^ | -1.6,-0.4 | -6.7^*^ | -6.9,-6.5 | -6.9^*^ | -7.2,-6.7 | -0.3^*^ | -0.4,-0.2 |
|  | 2010-2019 | -1.6^*^ | -2.0,-1.2 | -1.9^*^ | -2.1,-1.8 | -2.6^*^ | -4.2,-1.0 | -3.9^*^ | -4.6,-3.3 | -1.6^*^ | -1.6,-1.5 |

**Note:** Ischemic heart disease (IHD), ischemic stroke (IS), average annual percent change (AAPC), *: statistically significant (p < 0.05).

**Table S6:** The temporal trend of the age-standardized DALYs rate of IHD and IS attributed to modifiable dietary risk factor in the female population of China, Japan, South Korea, North Korea, and the world (1990-1999, 2000-2009, 2010-2019)

| DALYs |  | World | | China | | Japan | | South Korea | | North Korea | |
| --- | --- | --- | --- | --- | --- | --- | --- | --- | --- | --- | --- |
| Female | Trends | AAPC | 95%CI | AAPC | 95%CI | AAPC | 95%CI | AAPC | 95%CI | AAPC | 95%CI |
| IHD | 1990-1999 | -1.2^*^ | -1.5,-0.9 | -1.8^*^ | -2.2,-1.5 | -5.4^*^ | -5.6,-5.3 | -9.1^*^ | -9.5,-8.7 | 1.7^*^ | 1.6,1.8 |
|  | 2000-2009 | -1.8^*^ | -1.9,-1.8 | 0.5^*^ | 0.1,0.9 | -3.9^*^ | -4.0,-3.8 | -5.2^*^ | -5.4,-5.1 | 1.0^*^ | 0.8,1.3 |
|  | 2010-2019 | -1.2^*^ | -1.5,-1.0 | -1.3^*^ | -1.5,-1.2 | -2.4^*^ | -2.9,-2.0 | -2.8^*^ | -3.4,-2.2 | -1.3^*^ | -1.5,-1.2 |
| IS | 1990-1999 | -1.3^*^ | -1.5,-1.1 | -1.1^*^ | -1.7,-0.5 | -4.5^*^ | -5.1,-3.9 | -2.1^*^ | -2.5,-1.8 | 0.5^*^ | 0.4,0.6 |
|  | 2000-2009 | -2.3^*^ | -2.5,-2.1 | -0.7^*^ | -1.3,-0.1 | -5.4^*^ | -5.6,-5.1 | -6.4^*^ | -6.7,-6.2 | -0.4^*^ | -0.4,-0.3 |
|  | 2010-2019 | -1.0^*^ | -1.4,-0.7 | -1.3^*^ | -1.5,-1.1 | -1.6^*^ | -2.0,-1.2 | -3.7^*^ | -4.3,-3.1 | -1.2^*^ | -1.2,-1.1 |

**Note:** Disability-adjusted life years (DALYs), ischemic heart disease (IHD), ischemic stroke (IS), average annual percent change (AAPC), *: statistically significant (p < 0.05).

**Table S7:** The temporal trend of the mortality and DALYs rate of IHD and IS attributed to modifiable dietary risk factor in male and female of aged group (50-54 years) in China, Japan, South Korea, North Korea, and the world (1990-2019)

| Mortality |  | World | | China | | Japan | | South Korea | | North Korea | |
| --- | --- | --- | --- | --- | --- | --- | --- | --- | --- | --- | --- |
|  |  | AAPC | 95%CI | AAPC | 95%CI | AAPC | 95%CI | AAPC | 95%CI | AAPC | 95%CI |
| IHD | Male | -1.3^*^ | -1.5,-1.1 | -0.4 | -0.9,0.0 | -1.5^*^ | -1.7,-1.3 | -5.8^*^ | -6.1,-5.6 | 0.3^*^ | 0.2,0.3 |
|  | Female | -1.2^*^ | -1.4,-1.0 | -2.3^*^ | -2.8,-1.9 | -2.7^*^ | -2.8,-2.5 | -8.3^*^ | -8.7,-7.9 | 0.5^*^ | 0.4,0.6 |
| IS | Male | -1.3^*^ | -1.6,-1.0 | -1.1^*^ | -1.4,-0.8 | -3.9^*^ | -4.2,-3.6 | -5.9^*^ | -6.5,-5.3 | -0.9^*^ | -1.0,-0.8 |
|  | Female | -2.0^*^ | -2.6,-1.3 | -2.9^*^ | -3.4,-2.3 | -5.1^*^ | -5.6,-4.6 | -8.3^*^ | -9.5,-7.1 | -0.6^*^ | -0.7,0.6 |
| DALYs |  |  |  |  |  |  |  |  |  |  |  |
| IHD | Male | -1.3^*^ | -1.5,-1.1 | -0.4 | -0.8,0.0 | -1.5^*^ | -1.7,-1.3 | -5.7^*^ | -6.0,-5.5 | 0.3^*^ | 0.2,0.3 |
|  | Female | -1.1^*^ | -1.3,-0.9 | -2.1^*^ | -2.6,-1.7 | -2.6^*^ | -2.7,-2.5 | -8.0^*^ | -8.4,-7.7 | 0.5^*^ | 0.4,0.5 |
| IS | Male | -1.1^*^ | -1.4,-0.8 | -0.8^*^ | -1.0,-0.6 | -2.2^*^ | -2.4,-2.0 | -4.4^*^ | -4.6,-4.1 | -0.9^*^ | -1.0,-0.8 |
|  | Female | -1.3^*^ | -1.8,-0.9 | -1.8^*^ | -2.4,-1.2 | -1.8^*^ | -2.1,-1.5 | -4.0^*^ | -4.6,-3.4 | -0.6^*^ | -0.6,-0.5 |

**Note:** Disability-adjusted life years (DALYs), ischemic heart disease (IHD), ischemic stroke (IS), average annual percent change (AAPC), *: statistically significant (p < 0.05).

**Table S8:** The temporal trend of the mortality and DALYs rate of IHD and IS attributed to modifiable dietary risk factor in male and female of aged group (55-59 years) in China, Japan, South Korea, North Korea, and the world (1990-2019)

| Mortality |  | World | | China | | Japan | | South Korea | | North Korea | |
| --- | --- | --- | --- | --- | --- | --- | --- | --- | --- | --- | --- |
|  |  | AAPC | 95%CI | AAPC | 95%CI | AAPC | 95%CI | AAPC | 95%CI | AAPC | 95%CI |
| IHD | Male | -1.2^*^ | -1.5,-1.0 | -0.5^*^ | -0.9,-0.1 | -2.3^*^ | -2.6,-2.0 | -5.6^*^ | -5.9,-5.2 | 0.4^*^ | 0.3,0.4 |
|  | Female | -1.4^*^ | -1.6,-1.2 | -1.9^*^ | -3.1,-0.7 | -3.9^*^ | -4.1,-3.8 | -7.7^*^ | -8.2,-7.3 | 0.6^*^ | 0.5,0.7 |
| IS | Male | -1.4^*^ | -1.8,-1.1 | -0.9^*^ | -1.2,-0.6 | -4.7^*^ | -5.0,-4.5 | -6.1^*^ | -6.9,-5.3 | -0.5^*^ | -0.6,-0.4 |
|  | Female | -2.2^*^ | -3.2,-1.1 | -2.6^*^ | -3.8,-1.3 | -5.7^*^ | -6.5,-4.9 | -8.5^*^ | -9.9,-7.0 | -0.4^*^ | -0.5,-0.4 |
| DALYs |  |  |  |  |  |  |  |  |  |  |  |
| IHD | Male | -1.2^*^ | -1.4,-1.0 | -0.5^*^ | -0.9,-0.1 | -2.3^*^ | -2.6,-2.0 | -5.4^*^ | -5.7,-5.1 | 0.4^*^ | 0.3,0.4 |
|  | Female | -1.3^*^ | -1.5,-1.1 | -1.7^*^ | -2.8,-0.6 | -3.8^*^ | -3.9,-3.6 | -7.5^*^ | -7.9,-7.0 | 0.6^*^ | 0.5,0.7 |
| IS | Male | -1.2^*^ | -1.5,-0.9 | -0.6^*^ | -0.9,-0.4 | -3.1^*^ | -3.5,-2.7 | -4.7^*^ | -5.1,-4.2 | -0.5^*^ | -0.6,-0.4 |
|  | Female | -1.7^*^ | -2.0,-1.4 | -1.6^*^ | -2.4,-0.8 | -2.6^*^ | -2.8,-2.3 | -4.6^*^ | -5.2,-3.9 | -0.4^*^ | -0.5,-0.4 |

**Note:** Disability-adjusted life years (DALYs), ischemic heart disease (IHD), ischemic stroke (IS), average annual percent change (AAPC), *: statistically significant (p < 0.05).

**Table S9:** The temporal trend of the mortality and DALYs rate of IHD and IS attributed to modifiable dietary risk factor in male and female of aged group (60-64 years) in China, Japan, South Korea, North Korea, and the world (1990-2019)

| Mortality |  | World | | China | | Japan | | South Korea | | North Korea | |
| --- | --- | --- | --- | --- | --- | --- | --- | --- | --- | --- | --- |
|  |  | AAPC | 95%CI | AAPC | 95%CI | AAPC | 95%CI | AAPC | 95%CI | AAPC | 95%CI |
| IHD | Male | -1.5^*^ | -1.7,-1.3 | -0.2 | -0.7,0.3 | -2.8^*^ | -3.0,-2.6 | -6.0^*^ | -6.3,-5.6 | 0.4^*^ | 0.4,0.5 |
|  | Female | -1.6^*^ | -1.8,-1.4 | -1.3^*^ | -2.1,-0.5 | -4.5^*^ | -4.7,-4.3 | -7.1^*^ | -7.8,-6.4 | 0.5^*^ | 0.5,0.6 |
| IS | Male | -1.5^*^ | -1.8,-1.2 | -0.6^*^ | -0.9,-0.4 | -4.4^*^ | -4.9,-3.8 | -6.8^*^ | -7.6,-5.9 | -0.5^*^ | -0.5,-0.4 |
|  | Female | -2.2^*^ | -2.5,-1.9 | -1.9^*^ | -2.2,-1.6 | -6.1^*^ | -6.6,-5.7 | -8.8^*^ | -10.1,-7.4 | -0.5^*^ | -0.5,-0.4 |
| DALYs |  |  |  |  |  |  |  |  |  |  |  |
| IHD | Male | -1.5^*^ | -1.7,-1.3 | -0.2 | -0.7,0.3 | -2.8^*^ | -2.9,-2.6 | -5.8^*^ | -6.1,-5.5 | 0.4^*^ | 0.4,0.5 |
|  | Female | -1.6^*^ | -1.7,-1.4 | -1.2^*^ | -2.0,-0.4 | -4.4^*^ | -4.5,-4.2 | -7.8^*^ | -8.4,-7.2 | 0.5^*^ | 0.4,0.6 |
| IS | Male | -1.3^*^ | -1.6,-1.0 | -0.4^*^ | -0.6,-0.2 | -3.3^*^ | -3.8,-2.7 | -5.6^*^ | -6.1,-5.1 | -0.4^*^ | -0.5,-0.4 |
|  | Female | -1.7^*^ | -1.9,-1.4 | -1.2^*^ | -1.5,-0.8 | -3.4^*^ | -3.7,-3.1 | -5.6^*^ | -6.2,-5.0 | -0.4^*^ | -0.5,-0.4 |

**Note:** Disability-adjusted life years (DALYs), ischemic heart disease (IHD), ischemic stroke (IS), average annual percent change (AAPC), *: statistically significant (p < 0.05).

**Table S10:** The temporal trend of the mortality and DALYs rate of IHD and IS attributed to modifiable dietary risk factor in male and female of aged group (65-69 years) in China, Japan, South Korea, North Korea, and the world (1990-2019)

| Mortality |  | World | | China | | Japan | | South Korea | | North Korea | |
| --- | --- | --- | --- | --- | --- | --- | --- | --- | --- | --- | --- |
|  |  | AAPC | 95%CI | AAPC | 95%CI | AAPC | 95%CI | AAPC | 95%CI | AAPC | 95%CI |
| IHD | Male | -1.6^*^ | -1.9,-1.3 | -0.3 | -0.9,0.3 | -3.4^*^ | -3.5,-3.2 | -5.9^*^ | -6.2,-5.5 | 0.5^*^ | 0.5,0.6 |
|  | Female | -1.7^*^ | -2.1,-1.4 | -0.7^*^ | -1.5,-0.0 | -4.9^*^ | -5.1,-4.7 | -6.6^*^ | -7.3,-6.0 | 0.6^*^ | 0.6,0.7 |
| IS | Male | -1.3^*^ | -1.8,-0.9 | -0.5^*^ | -0.8,-0.1 | -5.3^*^ | -6.0,-4.6 | -7.3^*^ | -8.0,-6.6 | -0.2^*^ | -0.2,-0.1 |
|  | Female | -2.2^*^ | -2.8,-1.6 | -1.6^*^ | -2.0,-1.2 | -6.8^*^ | -7.6,-6.1 | -8.2^*^ | -9.3,-7.0 | -0.3^*^ | -0.3,-0.2 |
| DALYs |  |  |  |  |  |  |  |  |  |  |  |
| IHD | Male | -1.6^*^ | -1.8,-1.3 | -0.3 | -0.9,0.3 | -3.3^*^ | -3.5,-3.2 | -5.8^*^ | -6.1,-5.4 | 0.5^*^ | 0.5,0.6 |
|  | Female | -1.7^*^ | -2.0,-1.4 | -0.7^*^ | -1.4,-0.0 | -4.7^*^ | -4.9,-4.5 | -6.4^*^ | -7.1,-5.7 | 0.6^*^ | 0.6,0.7 |
| IS | Male | -1.2^*^ | -1.6,-0.8 | -0.2 | -0.5,0.0 | -4.3^*^ | -4.8,-3.7 | -6.2^*^ | -6.6,-5.7 | -0.1^*^ | -0.2,-0.1 |
|  | Female | -1.7^*^ | -2.2,-1.2 | -0.9^*^ | -1.1,-0.6 | -4.1^*^ | -4.6,-3.7 | -5.7^*^ | -6.3,-5.2 | -0.2^*^ | -0.2,-0.2 |

**Note:** Disability-adjusted life years (DALYs), ischemic heart disease (IHD), ischemic stroke (IS), average annual percent change (AAPC), *: statistically significant (p < 0.05).

**Table S11:** The temporal trend of the mortality and DALYs rate of IHD and IS attributed to modifiable dietary risk factor in male and female of aged group (70-74 years) in China, Japan, South Korea, North Korea, and the world (1990-2019)

| Mortality |  | World | | China | | Japan | | South Korea | | North Korea | |
| --- | --- | --- | --- | --- | --- | --- | --- | --- | --- | --- | --- |
|  |  | AAPC | 95%CI | AAPC | 95%CI | AAPC | 95%CI | AAPC | 95%CI | AAPC | 95%CI |
| IHD | Male | -1.6^*^ | -1.8,-1.5 | 0.2 | -0.3,0.8 | -3.8^*^ | -3.9,-3.7 | -5.7^*^ | -5.9,-5.5 | 0.6^*^ | 0.5,0.7 |
|  | Female | -1.8^*^ | -1.9,-1.6 | -0.0 | -0.6,0.6 | -5.2^*^ | -5.5,-4.9 | -6.2^*^ | -6.7,-5.7 | 0.5^*^ | 0.4,0.6 |
| IS | Male | -1.4^*^ | -1.6,-1.2 | -0.3^*^ | -0.6,-0.1 | -5.5^*^ | -6.0,-5.0 | -6.5^*^ | -7.2,-5.7 | -0.1^*^ | -0.1,-0.0 |
|  | Female | -2.2^*^ | -2.4,-1.9 | -1.3^*^ | -1.6,-1.0 | -7.4^*^ | -7.8,-7.0 | -7.1^*^ | -8.0,-6.2 | -0.5^*^ | -0.5,-0.4 |
| DALYs |  |  |  |  |  |  |  |  |  |  |  |
| IHD | Male | -1.6^*^ | -1.8,-1.5 | 0.2 | -0.3,0.7 | -3.7^*^ | -3.8,-3.6 | -5.6^*^ | -5.8,-5.4 | 0.6^*^ | 0.5,0.7 |
|  | Female | -1.7^*^ | -1.9,-1.6 | -0.1 | -0.6,0.5 | -5.0^*^ | -5.2,-4.7 | -6.1^*^ | -6.6,-5.6 | 0.6^*^ | 0.5,0.6 |
| IS | Male | -1.2^*^ | -1.4,-1.1 | -0.1 | -0.3,0.1 | -4.6^*^ | -5.0,-4.2 | -5.9^*^ | -6.3,-5.4 | -0.0^*^ | -0.1,-0.0 |
|  | Female | -1.8^*^ | -2.1,-1.5 | -0.7^*^ | -0.9,-0.5 | -5.1^*^ | -5.4,-4.8 | -5.9^*^ | -6.5,-5.2 | -0.3^*^ | -0.4,-0.3 |

**Note:** Disability-adjusted life years (DALYs), ischemic heart disease (IHD), ischemic stroke (IS), average annual percent change (AAPC), *: statistically significant (p < 0.05).

**Table S12:** The temporal trend of the mortality and DALYs rate of IHD and IS attributed to modifiable dietary risk factor in male and female of aged group (75-79 years) in China, Japan, South Korea, North Korea, and the world (1990-2019)

| Mortality |  | World | | China | | Japan | | South Korea | | North Korea | |
| --- | --- | --- | --- | --- | --- | --- | --- | --- | --- | --- | --- |
|  |  | AAPC | 95%CI | AAPC | 95%CI | AAPC | 95%CI | AAPC | 95%CI | AAPC | 95%CI |
| IHD | Male | -1.6^*^ | -1.9,-1.4 | 0.6^*^ | 0.1,1.2 | -4.0^*^ | -4.2,-3.9 | -5.3^*^ | -5.5,-5.1 | 0.7^*^ | 0.5,0.8 |
|  | Female | -1.8^*^ | -2.1,-1.6 | 0.3 | -0.5,1.2 | -5.3^*^ | -5.6,-5.1 | -5.4^*^ | -5.9,-5.0 | 0.5^*^ | 0.4,0.6 |
| IS | Male | -1.3^*^ | -1.5,-1.2 | -0.2 | -0.5,-0.2 | -5.8^*^ | -6.3,-5.3 | -5.8^*^ | -6.4,-5.2 | 0.0 | -0.0,0.1 |
|  | Female | -2.2^*^ | -2.5,-2.0 | -0.9^*^ | -1.2,-0.5 | -7.7^*^ | -8.1,-7.2 | -6.0^*^ | -6.8,-5.2 | -0.6^*^ | -0.6,-0.5 |
| DALYs |  |  |  |  |  |  |  |  |  |  |  |
| IHD | Male | -1.6^*^ | -1.9,-1.4 | 0.6^*^ | 0.0,1.1 | -3.9^*^ | -4.1,-3.8 | -5.3^*^ | -5.5,-5.1 | 0.6^*^ | 0.5,0.7 |
|  | Female | -1.8^*^ | -2.0,-1.6 | 0.3 | -0.5,1.1 | -5.1^*^ | -5.4,-4.9 | -5.3^*^ | -5.8,-4.9 | 0.5^*^ | 0.4,0.6 |
| IS | Male | -1.2^*^ | -1.3,-1.1 | 0.0 | -0.3,0.4 | -5.0^*^ | -5.4,-4.6 | -5.2^*^ | -5.7,-4.8 | 0.0 | -0.0,0.1 |
|  | Female | -1.9^*^ | -2.1,-1.7 | -0.4^*^ | -0.7,-0.1 | -5.6^*^ | -5.9,-5.3 | -5.2^*^ | -5.8,-4.6 | -0.4^*^ | -0.5,-0.4 |

**Note:** Disability-adjusted life years (DALYs), ischemic heart disease (IHD), ischemic stroke (IS), average annual percent change (AAPC), *: statistically significant (p < 0.05).

**Table S13: The ranking of dietary risk factors attributed to IHD mortality in the East Asian countries and world 2019.**

| Risk factors | World | China | Japan | South Korea | North Korea |
| --- | --- | --- | --- | --- | --- |
| Mortality | ASR (95% UL) | ASR (95% UL) | ASR (95% UL) | ASR (95% UL) | ASR (95% UL) |
| Diet low in whole grains | 18.6 (24.4,7.0) | 17.5 (24.0,6.1) | 4.2 (5.8,1.5) | 4.0 (5.8,1.5) | 17.2 (24.3,6.3) |
| Diet low in legumes | 14.3 (23.1,3.3) | 12.3 (20.6,1.8) | 1.9 (3.4,0.2) | 4.7 (7.7,1.2) | 12.0 (20.8,1.5) |
| Diet high in sodium | 9.8 (22.6,2.3) | 16.9 (32.4,5.9) | 2.8 (6.7,0.4) | 4.6 (9.8,1.0) | 17.2 (33.9,4.4) |
| Diet high in trans fatty acids | 8.2 (11.3,1.0) | 6.5 (9.3,0.7) | 1.6 (2.5,0.3) | 3.2 (4.5,0.2) | 6.3 (10.7,1.2) |
| Diet low in nuts and seeds | 7.0 (9.7,3.6) | 5.2 (8.3,1.9) | 2.6 (3.6,1.3) | 1.7 (2.6,0.7) | 14.2 (19.6,9.7) |
| Diet low in fruits | 5.5 (8.4,2.3) | 4.3 (7.0,1.4) | 1.5 (2.3,0.6) | 1.4 (2.2,0.5) | 6.1 (9.7,11.9) |
| Diet low in vegetables | 4.5 (6.5,2.5) | 0.6 (1.0,0.3) | 0.4 (0.8,0.1) | 1.0 (1.5,0.4) | 5.1 (8.1,1.8) |
| Diet low in polyunsaturated fatty acids | 4.5 (8.3,0.7) | 4.6 (9.6,0.7) | 0.8 (1.7,0.1) | 0.4 (0.9,0.1) | 2.9 (6.4,0.6) |
| Diet low in fiber | 4.5 (7.3,1.9) | 2.3 (4.3,0.8) | 1.3 (2.2,0.5) | 3.4 (5.1,1.7) | 7.2 (12.1,2.6) |
| Diet high in red meat | 4.4 (9.0,0.6) | 5.8 (11.1,0.8) | 0.9 (1.9,0.1) | 1.4 (2.7,0.2) | 2.7 (5.6,0.3) |
| Diet low in seafood omega-3 fatty acids | 4.3 (5.6,2.1) | 4.3 (5.9,2.0) | 1.0 (1.7,0.5) | 1.2 (1.5,0.6) | 4.8 (7.1,1.6) |
| Diet high in processed meat | 2.5 (4.9,0.6) | 0.9 (2.3,0.3) | 1.1 (2.2,0.1) | 0.6 (1.5,0.1) | 0.9 (2.4,0.3) |
| Diet high in sugar-sweetened beverages | 2.5 (3.2,1.7) | 2.7 (3.6,1.9) | 0.6 (0.9,0.3) | 0.6 (0.9,0.3) | 4.0 (5.5,2.9) |

**Note:** Ischemic heart disease (IHD), age-standardized rate (ASR) per 100,000 persons, upper (U), lower (L)

**Table S14: The ranking of dietary risk factors attributed to IHD DALYs in the East Asian countries and world 2019.**

| Risk factors | World | China | Japan | South Korea | North Korea |
| --- | --- | --- | --- | --- | --- |
| DALYs | ASR (95% UL) | ASR (95% UL) | ASR (95% UL) | ASR (95% UL) | ASR (95% UL) |
| Diet low in whole grains | 393.6 (511.2,148.9) | 320.7 (438.1,110.0) | 86.4 (115.5,31.5) | 64.7 (92.8,25.1) | 382.8 (551.7,142.4) |
| Diet low in legumes | 297.5 (483.8,64.5) | 218.3 (367.4,32.3) | 38.8 (67.5,4.2) | 73.2 (122.3,15.5) | 260.9 (462.9,26.7) |
| Diet high in sodium | 210.4 (449.3,56.5) | 352.2 (607.0,152.1) | 52.9 (119.5,7.0) | 70.5 (144.2,15.3) | 366.6 (695.5,104.1) |
| Diet high in trans fatty acids | 173.9 (237.3,19.4) | 115.0 (164.8,11.8) | 29.9 (46.6,5.4) | 52.5 (71.9,3.3) | 132.0 (222.7,24.8) |
| Diet low in nuts and seeds | 150.0 (206.7,77.7) | 88.1 (141.5,34.1) | 49.0 (68.0,23.0) | 20.2 (33.5,7.2) | 307.6 (429.3,199.9) |
| Diet low in fruits | 124.6 (184.5,51.6) | 78.3 (125.0,25.3) | 32.2 (48.2,13.0) | 21.5 (34.2,6.9) | 136.2 (220.7,43.9) |
| Diet low in vegetables | 102.1 (145.7,56.8) | 7.6 (12.6,4.2) | 8.3 (14.2,2.3) | 10.7 (17.5,3.9) | 108.4 (177.0,36.8) |
| Diet low in polyunsaturated fatty acids | 97.6 (198.0,11.9) | 82.9 (171.8,11.4) | 15.6 (34.2,1.9) | 5.8 (13.6,1.4) | 62.2 (140.4,10.6) |
| Diet low in fiber | 97.4 (158.9,41.4) | 44.9 (82.5,16.0) | 27.0 (44.4,11.2) | 51.1 (77.0,23.9) | 156.1 (267.0,56.4) |
| Diet high in red meat | 94.6 (172.3,16.3) | 116.5 (216.1,21.1) | 20.4 (40.5,1.3) | 28.2 (53.5,4.5) | 63.6 (135.9,5.2) |
| Diet low in seafood omega-3 fatty acids | 90.6 (118.4,41.5) | 75.9 (104.7,34.7) | 14.5 (28.4,5.5) | 18.8 (24.6,10.4) | 104.3 (159.7,33.3) |
| Diet high in processed meat | 50.8 (98.3,12.2) | 17.9 (48.8,4.3) | 28.6 (53.9,3.4) | 11.1 (29.3,1.2) | 21.4 (60.5,4.9) |
| Diet high in sugar-sweetened beverages | 48.9 (64.1,30.5) | 41.8 (55.4,29.7) | 12.0 (16.9,5.9) | 9.2 (13.7,4.0) | 76.2 (102.2,54.5) |

**Note:** Ischemic heart disease (IHD), disability-adjusted life years (DALYs), age-standardized rate (ASR) per 100,000 persons, upper (U), lower (L)

**Table S15: The ranking of dietary risk factors attributed to IS mortality and DALYs in the East Asian countries and world 2019.**

| Risk factors | World | China | Japan | South Korea | North Korea |
| --- | --- | --- | --- | --- | --- |
| Mortality | ASR (95% UL) | ASR (95% UL) | ASR (95% UL) | ASR (95% UL) | ASR (95% UL) |
| Diet high in sodium | 3.7 (8.0,0.9) | 8.2 (15.7,2.7) | 1.2 (3.0,0.1) | 3.1 (6.8,0.6) | 8.5 (17.0,2.2) |
| Diet high in red meat | 2.2 (3.0,1.0) | 4.1 (5.8,2.0) | 0.6 (1.1,0.2) | 1.5 (2.5,0.5) | 2.7 (5.4,0.6) |
| Diet low in whole grains | 1.8 (2.7,0.6) | 2.5 (3.8,0.6) | 0.5 (0.8,0.1) | 1.2 (1.8,0.4) | 2.5 (4.0,0.5) |
| Diet low in fruits | 1.6 (2.9,0.4) | 2.0 (3.9,0.5) | 0.5 (0.7,0.1) | 0.9 (1.8,0.2) | 2.5 (4.7,0.6) |
| Diet low in fiber | 1.1 (1.9,0.3) | 0.8 (1.7,0.2) | 0.4 (0.8,0.1) | 0.8 (1.3,0.2) | 1.3 (2.2,0.2) |
| Diet low in vegetables | 0.6 (1.1,0.1) | 0.2 (0.3,0.1) | 0.1 (0.2,0.0) | 0.3 (0.6,0.1) | 1.2 (2.3,0.2) |
| DALYs |  |  |  |  |  |
| Diet high in sodium | 82.9 (168.4,24.8) | 195.6 (341.5,76.3) | 28.0 (64.8,3.6) | 58.8 (119.9,12.2) | 206.4 (389.6,59.1) |
| Diet high in red meat | 50.1 (68.2,25.0) | 100.0 (135.2,53.2) | 16.3 (30.9,4.4) | 33.2 (46.9,15.5) | 69.1 (135.4,15.5) |
| Diet low in whole grains | 40.3 (58.8,12.1) | 54.7 (83.8,13.3) | 14.7 (22.5,3.4) | 29.9 (48.2,9.3) | 63.6 (99.9,13.5) |
| Diet low in fruits | 35.5 (65.5,10.0) | 43.7 (84.1,10.9) | 14.3 (22.2,3.8) | 18.4 (36.8,4.3) | 61.4 (113.7,14.1) |
| Diet low in fiber | 23.1 (40.6,6.5) | 19.6 (39.2,5.0) | 11.3 (20.5,2.8) | 17.2 (26.9,3.2) | 34.4 (61.4,4.6) |
| Diet low in vegetables | 12.8 (23.1,3.0) | 3.1 (4.7,2.2) | 2.5 (5.0,0.7) | 4.6 (9.1,1.1) | 28.7 (56.9,3.2) |

**Note:** Ischemic stroke (IS), disability-adjusted life years (DALYs), age-standardized rate (ASR) per 100,000 persons, upper (U), lower (L)
