# Supplementary figures and images for: Prediction of cardiovascular diseases mortality- and disability-adjusted life-years attributed to modifiable dietary risk factors from 1990 to 2030 among East Asian countries and the world
Source: Front Nutr. 2022 Oct 17;9:898978. doi: 10.3389/fnut.2022.898978 (PMC9618868; doi:10.3389/fnut.2022.898978)

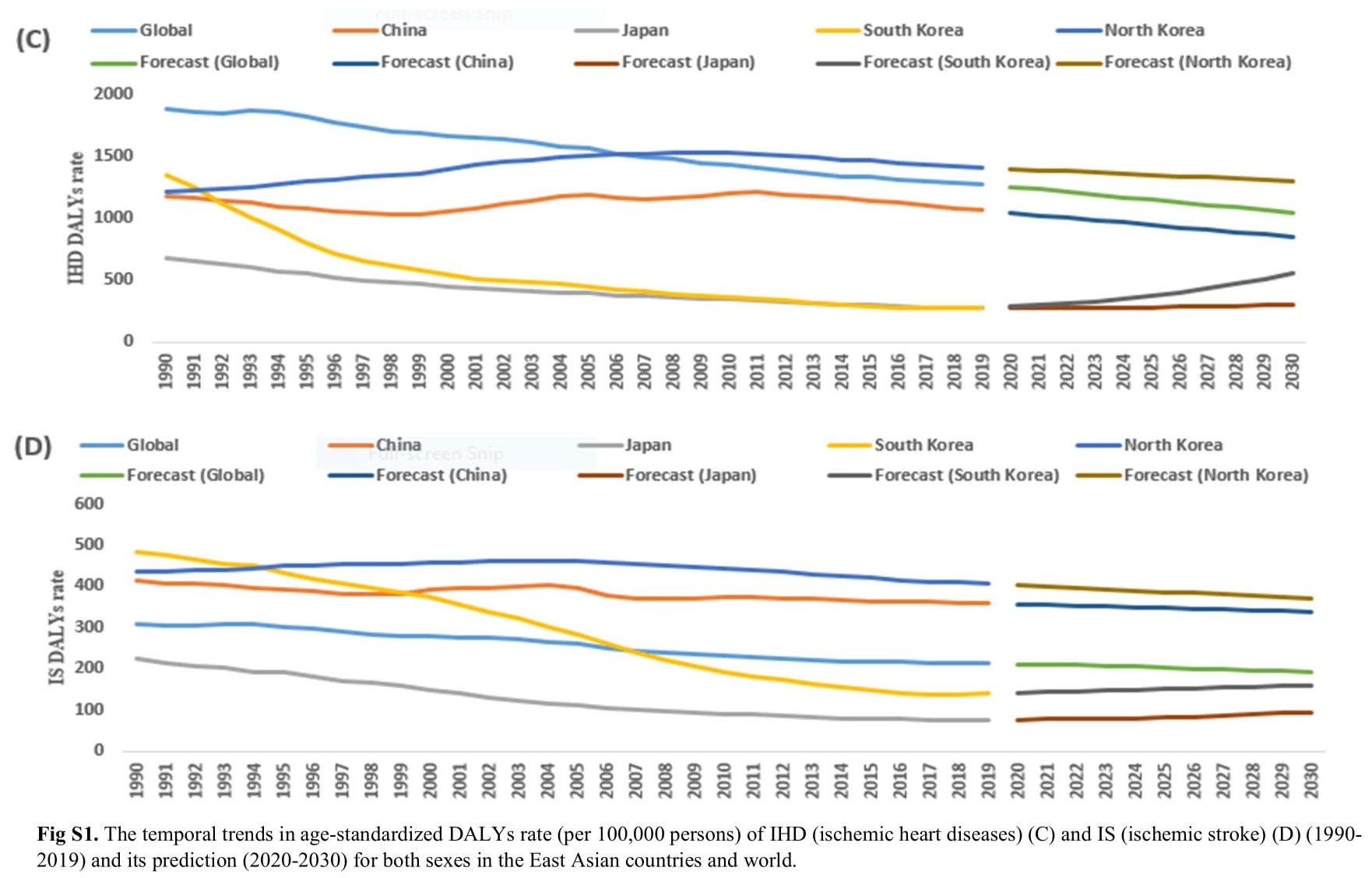


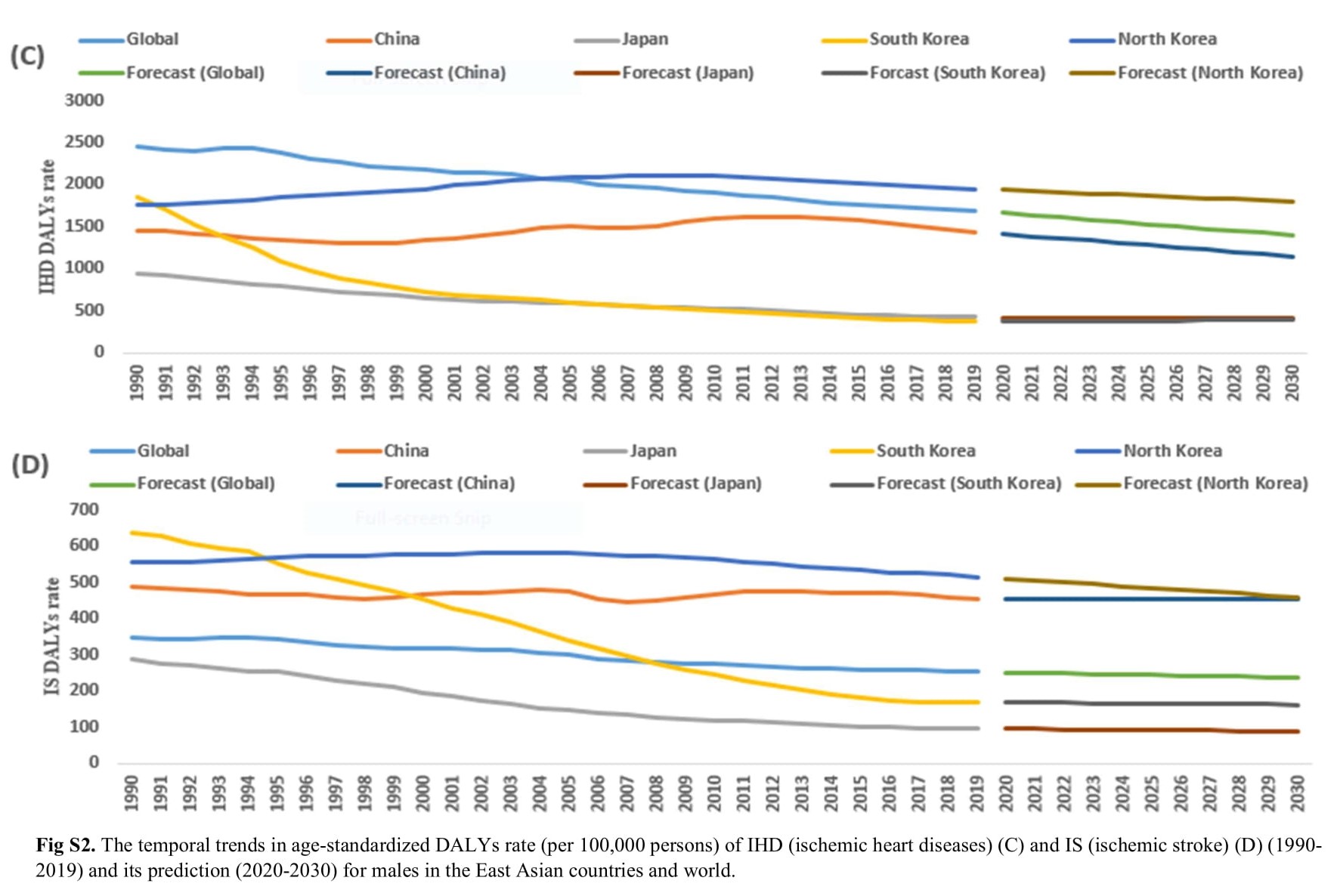


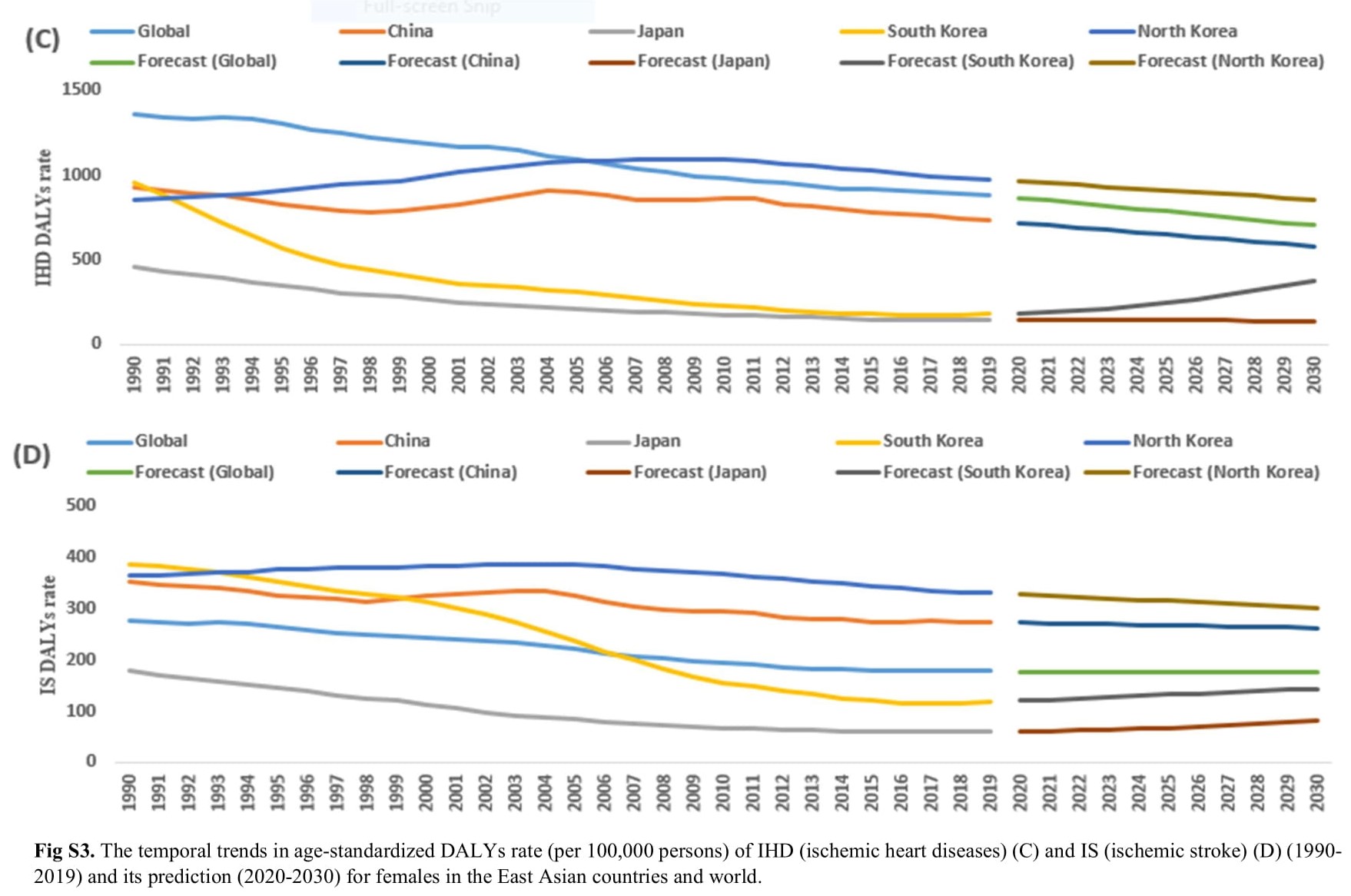

Supplement: Supplementary file 2 [file Data_Sheet_2.docx]
